# Report of a Case of Chronic Diarrhœa, with Muco-Sanguineous Discharges, Depending upon Ulceration of the Rectum

**Published:** 1864-08

**Authors:** Robert Taylor

**Affiliations:** Pendleton, Ohio


ART. III.—*Report of a case of Chronic Diarrhœa, with Muco-Sanguineous discharges, depending upon Ulceration of the Rectum. By* ROBERT TAYLOR, M. D., *Pendleton, Ohio, July* 15*th*, 1864.

J. H., aged fifty-five, private in 57th Regiment O. V. I., of stout build, robust constitution; health good for eighteen months after entering the service, when he was attacked with what is commonly designated Camp Diarrhœa; at first simple in its character, but gradually assuming the chronic form.

Being improperly treated, or not treated at all, the diarrhœa ran along unchecked from bad to worse. At the end of three months, bloody discharges intervened, at times preceding the fæces, at other times being wholly muco-sanguinolent, never intermingled.

One month more he was removed to a field hospital, where he remained under treatment six weeks, at times improving, again becoming worse.

On October 1st, 1863, he was admitted to the General Hospital at Memphis, Tennesee, where, from the remarks as to the condition of the patient, copied from the medical descriptive list, I find he was greatly emaciated, anæmic, and exhausted, with occasional pains in the abdomen, and fever of an irregular intermittent type. Nervous twitchings at times manifested themselves with oedema of the lower extremities, a result of the diarrhœa and sanguinolent discharges, depriving the blood of its albumen and corpuscles, thus rendering it more serous—hence the anasarca.

Here he was treated with a great variety of therapeutical agents, simply for the diarrhœa, no inquiry being made as to the cause of the bloody discharges, probably referring it to internal hæmorrhoidal tumors, as the patient himself was under this impression when I first saw him. After remaining a long time in this hospital, and practically exhausting the list of astringents and anti-diarrhœa remedies laid down in materia medica to little or no avail, he was discharged from service and his pension certificate afterward, signed by the examining surgeon for pensions at St. Louis, Mo.

Perhaps it would not be improper to add, that the last remarks made in the descriptive list by the surgeon in charge at Memphis was, *"diarrhœa more frequent, patient worse."*

May 12th.—Stimulated by the thoughts of home and friends, the old veteran through great exertion had completed the journey to Western Ohio, where I was requested to visit him.

He had entered the service through patriotic motives alone, not being compelled to do so on account of limited means, as he was a wealthy farmer. He was very anxious to get well, that he might see the rebellion put down. He was also willing to submit or do anything I might think proper for his relief.

The general conditions of the patient, were but little changed from that described while at Memphis, only all the symptoms being somewhat aggravated. The whole number of discharges averaged from twenty to thirty per day, of which nearly one-third consisted of blood and mucous. Upon Inquiry I ascertained the patient never had any symptoms of hæmorrhoids, previous to the first three months of his illness. This circumstance led me to suspect ulceration of the rectum, instead of piles' as had been previously supposed. I therefore made an examination by the aid of a speculum and found my diagnosis verified, as the entire mucous surface of the rectum was extensively ulcerated. Here then was a plain solution of the vexed problem, why the patient did not recover while at Memphis. The diarrœha having caused the ulceration of the rectum through the frequen tand long continued acrid discharges over its mucus surface; while the ulceration in turn, caused the continuation of the diarrhœa through sympathy.

It was now quite evident that if the cause could be removed, (to-wit: the ulceration,) a cure might be effected. The extent of the ulcerated surface, could not, however, be fully ascertained, as it apparently extended beyond the reach of the speculum. Doubts were, therefore, entertained as to his final recovery, as local remedial agents were mainly to be relied upon to effect a cure of the ulcerated mucus membrane. The treatment adopted was both local and constitutional.

*Local Treatment.*—The ulcerated surface being cleansed with bits of cotton dossil, a solution of arg. nitras, gr. x, to aqua puræ ℥ i, was freely applied through a speculum by means of a swab, until all bloody exudation ceased, and the surface was thoroughly cauterized. This treatment was at first continued daily for eight successive days, when through the greatly improved condition of the parts, the application was made semi-weekly for near a month longer, small spots of disease however still remained, so that it was necessary to continue the application weekly for another month, after which local treatment was discontinued.

At the beginning the patient experienced but little pain from the cauterization, but after several applications sensation became quite manifest, the ulcerated surface assumed a healthier appearance, and the diarrhœa became gradually less frequent.

The conditions of the patient required a supportive and anti-diarrhœal, constitutional treatment. For the former I prescribed the following:

| | |
|---|---|
| ℞ Ferri Sul. - - - - | gr. ii. |
| Quinæ Sul. - - - - - | gr. i. |

Fiat pil. one every six hours.

For the latter,

| | |
|---|---|
| ℞ Pulvis Opii, - - - - | ʒ i. |
| Strychnia, - - - - - | gr. vi. |
| Arg Nitras, - - - - | ʒ ss. |

Fiat pillulæ 60, one every six hours, alternating with the powder.

This, particularly, seemed to suit the existing indications, as was fully proven by the patient rapidly improving under its administration.

Here let us pass in review a few of the properties of the above employed agents, so that we may appreciate their good qualities in a disease both frequent and formidable. You will remember that the patient was anæmic, with serous infiltration of the lower extremities, a result of a loss of albumen and corpuscles the blood had sustained, though a long continued sanguinous and diarrhœal discharge.

Quinia is well known by all, to be in small doses tonic, and having the power to increase albumen in the circulation.

Sulphate of iron is both tonic and astringent, and a restorer of the corpuscles of the blood. Here then were combined in one, properties to meet in part the two conditions.

The value of opium in diarrhœa is too well known and appreciated to need comment.

Strychnia is a tonic well adapted in chronic diarrhœa, and particularly applicable in this case, as nervous debility existed, manifesting itself by frequent muscular twitchings, etc.

Nitrate of silver is also a tonic, highly valuable in all irritable conditions of the mucous membrane of the stomach and bowels.

The above treatment was mostly adhered to, throughout the entire time needed for the removal of the disease, and with the best result, convalescence commenced early and continued gradually until a permanent cure was accomplished.

I have omitted full daily notes of the progress, symptoms, and slight changes of treatment, necessary at certain times, as it would be unnecessarily lengthy, besides there was but little change of symptoms, aside from a sure, gradual, and permanent recovery.

In concluding these abbreviated notes, I have only to add, that my object in their publication, was simply to call attention to a disease of frerquent occurrence, and well known to every one, yet a disease of great fatality, especially among our troops in various localities and departments; a disease, the proper treatment of which I fear is often greatly overlooked or grossly neglected.

From various and reliable sources, I learn that a sanguinolent flux is concomitant to this affection among our soldiers, and that but little attention in many instances is paid thereto by the surgeon in charge. Hence we need not be surprised that death claims so many victims from an affection, and if properly treated generally amenable to cure.

